# Head and Neck Pain in Patients Presenting with Acute Aortic Dissection

**DOI:** 10.1055/s-0039-18388

**Published:** 2019-04-24

**Authors:** Stephen Philip, Emil Missov, Dan Gilon, Stuart Hutchison, Ali Khoynezhad, Arturo Evangelista, Mark Bonaca, Lori Conklin, Jehangir Appoo, Marco Di Eusanio, Alan Braverman, Alberto Forteza, Daniel Montgomery, Christoph Nienaber, Eric Isselbacher, Kim Eagle

**Affiliations:** 1 Department of Internal Medicine, University of Michigan Health System, Ann Arbor, Michigan; 2 Department of Internal Medicine, University of Minnesota Physicians Heart Practice, Minneapolis, Minnesota; 3 Department of Non-invasive Cardiology, Hadassah Hebrew University Medical Center, Jerusalem, Israel; 4 Libin Cardiovascular Institute, University of Calgary Medical Centre, Calgary, Canada; 5 Department of Cardiovascular Surgery, Long Beach Medical Center, Los Angeles, California; 6 Department of Cardiology, Hospital General Universitari Vall D'hebron, Barcelona, Spain; 7 Department of Cardiovascular Medicine, Brigham & Women's Hospital, Boston, Massachusetts; 8 Department of Anesthesiology, University of Virginia Health System, Charlottesville, Virginia; 9 Department of Cardiovascular Surgery, Ospedali Riuniti Ancona, Ancona, Italy; 10 Deparment of Cardiology, Washington University, School of Medicine, St. Louis, Missouri; 11 Department of Cardiac Surgery, Hospital Universitario Puerta De Hierro, Madrid, Spain; 12 Cardiology and Aortic Centre, The Royal Brompton & Harefield Nhs Trust, London, United Kingdom; 13 Thoracic Aortic Center, Massachusetts General Hospital, Boston, Massachusetts

**Keywords:** Head Pain, neck pain, aortic dissection

## Abstract

**Background** Head and neck pain is an atypical presentation of acute aortic dissection. Classic teaching associates this pain with proximal dissections, but this has not been extensively studied.

**Methods** Patients enrolled in the International Registry of Acute Aortic Dissection from January 1996 to March 2015 were included in this study. We analyzed the demographics, presentation, treatment, and outcomes of Type A aortic dissection patients presenting with head and neck pain ( *n* = 812, 25.8%) and compared it with those without these symptoms ( *n* = 2,341, 74.2%).

**Results** Patients with head and neck pain were more likely to be white, female, with a family history of aortic disease. Patients with head and neck pain had higher percentages of back pain (43.3% vs. 37.5%, *p* = 0.005) and chest pain (87.6% vs. 79.3%, *p* < 0.001). On imaging, a higher percentage of those with head and neck pain had arch vessel involvement (44.3% vs. 38%, *p* = 0.010) and intramural hematoma (11.7% vs. 8.1%, *p* = 0.003). Surgical management was more common in patients with head and neck pain (89.8% vs. 85.2%, *p* = 0.001). Regarding outcomes, patients with head and neck pain had significantly higher rates of stroke than those without head and neck pain (13% vs. 9.9%, *p* = 0.016); however, overall mortality was lower for those with head and neck pain (19.5% vs. 23%, *p* = 0.038). Those with head and neck pain only had higher overall mortality compared to those with head and neck pain with chest or back pain (34.6% vs. 19.9%, *p* = 0.013). A logistic regression of mortality revealed that preoperative hypotension and age > 65 years were significantly associated with increased mortality.

**Conclusion** Presence of head and neck pain in Type A dissection is associated with more arch involvement, intramural hematoma, and stroke. When isolating those with head and neck pain only, there appear to be a higher rate of comorbidity burden and higher overall mortality.

## Introduction

A 53-year-old Caucasian man was admitted to the hospital because of intense frontal headache and neck pain. In the past, he suffered from occasional migraine with aura, with predominant parietotemporal and occipital headache. The present episode started 36 hours before admission and was ushered in with a stabbing anterior chest pain lasting 10 minutes. A few minutes after the onset of chest pain, severe bifrontal headache evolved within 2 minutes, followed by additional neck pain. Moreover, the patient complained of nausea, dizziness, and flickering eyes. The headache persisted despite analgesics. Computed tomography scan showed no subarachnoid hemorrhage or aneurysm. Chest X-ray showed no abnormalities. However, transthoracic echocardiography showed a widened ascending aorta (54 mm) with an intimal flap and a severely regurgitant tricuspid aortic valve. 1

Acute aortic dissection is a highly lethal condition, which has an estimated incidence of 2.9 per 100,000 in western Hungary to 3.5 per 100,000 person-years in Olmsted County, Minnesota. 2 3 Though this condition is rare compared to myocardial infarction that has an incidence of 208 cases per 100,000 person-years, aortic dissection is still the most common life-threatening disorder affecting the aorta. 4 5 6 This acute syndrome is rapidly fatal if undetected with a 20% prehospital lethality rate and up to 30% in-hospital mortality. 7 8 9 The mortality reaches 1 to 3% per hour in the first 48 hours and without treatment, more than 75% of patients with a Type A dissection will die in 2 weeks. 10 One of the difficulties with aortic dissection is the myriad ways in which it can present. Work done by the International Registry of Acute Aortic Dissection (IRAD) has shown that though many patients present with sudden severe chest pain, pulse deficits, and pain radiation, 10 to 15% of patients do not present with pain and a subset of other patients present with nonclassic symptoms such as neurologic deficits and syncope. 4 8 11

These atypically presenting patients have a potential for delayed diagnosis, treatment, and worse outcomes compared with classically presenting patients. One of these atypical presentation symptoms is head and neck pain. Clinical teaching states that proximal dissections are more often associated with head and neck pain and neurologic symptoms due to arch involvement; however, there are little data in the published literature to support this claim. 12 We sought to analyze the truth of this clinical anecdote while also looking at the demographics, presentation, treatment, and outcomes for acute Type A dissection patients with head and neck pain.

## Materials and Methods

The patient's information regarding acute aortic dissection was collected from 43 centers in North America, Europe, Asia, and Australia on patients who presented between January 1996 and March 2015. These patients were identified via hospital discharge diagnosis records and the imaging databases of surgical and echocardiography laboratories. A 290-question survey, which included questions on demographics, history, physical findings, management, imaging, and outcomes, was then filled out. These data were then entered into an online database and reviewed by the IRAD coordinating center at the University of Michigan for face validity and completeness. 4

The 3,027 acute Type A dissection patients were first separated into two cohorts: those with head and neck pain (791 patients) and those without head and neck pain (2,236 patients) for our main analysis. These groups were then further analyzed regarding demographic characteristics, presentation signs and symptoms, treatment types, and outcomes. Given our results, we performed a subgroup study in patients with head and neck pain, separating that group into those who had chest and back pain versus those without chest and back pain (therefore only head and neck pain). We also performed a logistic regression of mortality in this subset controlling for head and neck pain status, preoperative hypotension, age > 65 years, site of intimal tear, and signs of intramural hematoma.

Categorical variables were analyzed using Pearson's chi-square analysis. Continuous variables without skewed means were analyzed using two-sided *t* -tests. Skewed continuous variables were analyzed using the Whitney-Mann U asymptotic test. Logistic regressions models were analyzed using Hosmer-Lemeshow tests. Wald chi-square tests were used to find significance of the predictors.

## Results

Our main analysis found several differences in the demographics between patients with head and neck pain versus those without head and neck pain as seen in Table 1 . There were significantly more females with head and neck pain compared with those without (39.8% vs. 31.3% *p* < 0.001). There were significantly more white patients with head and neck pain (90.2%) compared with those without (84%, *p* < 0.001). There were fewer African American patients with head and neck pain (4.9% vs. 8.1% *p* = 0.004) and fewer Asian patients with head and neck pain (2.3% vs. 5.3% *p* = 0.001). Finally, atherosclerosis and a family history of aortic disease were found more often in those with head and neck pain than those without (23.7% vs. 18.9% *p* = 0.005, and 14.5% vs. 8.8% *p* = 0.002, respectively).

**Table 1:** Comparison of baseline demographic risk factors in patients with head and neck pain compared with those without head and neck pain

Demographics	Head and neck pain group	No head and neck pain group	*p* -Value
Age (overall mean)	61.49	61.45	0.939
< 40 y	8.6	7.4	0.27
> 70 y	30	30.4	0.831
Female sex	39.8	31.3	**< 0.001**
White	90.2	84	**< 0.001**
African American	4.9	8.1	**0.004**
Asian	2.3	5.3	**0.001**
Family history of aortic disease	14.5	8.8	**0.002**
Risk factors:			
Marfan's syndrome	4.6	3.7	0.281
Smoking	53.7	51.9	0.549
Hypertension	72.6	72.5	0.965
Atherosclerosis	23.7	18.9	**0.005**
Cocaine usage	1.5	1.9	0.534
Bicuspid aortic valve	5.3	3.9	0.13
Diabetes	8.8	7.2	0.134
Prior surgery:			
Coronary artery bypass grafting	3.5	5.3	0.053
Aortic valve replacement	3.1	4.8	0.061
Aortic aneurysm/dissection repair	7	6.1	0.407

Note: Bold values indicate significance at *p* < 0.05.

Regarding presentation symptoms ( Table 2 ), there appeared to be no significant difference in time from symptom onset to diagnosis or treatment between the two groups. There were interesting patterns in the quality of pain, however. Those with head and neck pain described significantly more sharp pain (49.6% vs. 43.6% *p* = 0.024), pressure-type pain (50.4% vs. 38% *p* < 0.001), abrupt onset of pain (89.8% vs. 78.5% *p* < 0.001), back pain (43.3% vs. 37.5% *p* = 0.005), chest pain (87.6% vs. 79.3% *p* < 0.001), and migrating pain (20.1% vs. 11% *p* < 0.001). Patients with head and neck pain also tended to present with cardiovascular accident (CVA) more often (7% vs. 4.6% *p* = 0.01), arch vessel involvement (44.3% vs 38% *p* = 0.01), and intramural hematoma (11.7% vs. 8.1% *p* = 0.003). However, patients with head and neck pain had significantly lower rates of congestive heart failure (CHF) (4.8% vs. 7% *p* = 0.03) and shock and tamponade (8.3% vs. 11.1% *p* = 0.03).

**Table 2:** Comparison of presenting symptoms in patients with head and neck pain compared with those without head and neck pain

Variables	Head/neck pain group	No head/neck pain group	*p* -Value
Hours from symptom onset to presentation at initial hospital	0.75–3.33	0.833–3.5	0.189
Presenting within 6 h of symptom onset	87.4	84	0.076
Hours from symptom onset to presentation at initial hospital	0.75–3.33	0.833–3.5	0.189
Hours from symptom onset to diagnosis (avg)	2.87–12.75	2.66–12.19	0.534
Hours from symptom onset to diagnosis:			
0–4 h	40.6	41.9	
4–24 h	45.2	43.1	
24 h	14.2	15.1	
Quality of pain:			
Tearing/ripping	29.9	30.2	0.926
Sharp	49.6	43.6	**0.024**
Pressure	50.4	38	**< 0.001**
Burning	13.8	12.6	0.534
Abrupt onset of pain	89.8	78.5	**< 0.001**
Back pain	43.3	37.5	**0.005**
Chest pain	87.6	79.3	**< 0.001**
Migrating pain	20.1	11	**< 0.001**
Coma/altered consciousness	10	10.7	0.598
Syncope	16.7	16.5	0.927
Cardiovascular accident	7	4.6	**0.01**
Congestive heart failure	4.8	7	**0.03**
Hypotension/shock/tamponade	24.9	27.6	0.168
Shock/tamponade	8.3	11.1	**0.03**
Shock	5.7	7.8	0.07
Tamponade	2.5	3.4	0.266
Murmur of aortic insufficiency	35.8	33.7	0.337
Any pulse deficit	33	34.8	0.451
Diagnostic imaging findings:			
Arch vessel involvement	44.3	38	**0.01**
Intramural hematoma (def)	11.7	8.1	**0.003**
Periaortic hematoma	21	19.6	0.463
False lumen thrombosis, complete	9.3	9	0.855
False lumen thrombosis, partial	18.9	20.3	0.517
Coronary artery compromise	12.9	12.9	0.978
Pericardial effusion	41.9	41.9	0.993
Chest X-ray findings:			
% abnormal	72.8	73.3	0.82
Wide mediastinum	53	52.7	0.931
Abnormal aortic contour	41.6	41.8	0.922
Abnormal cardiac contour	22.6	24.8	0.362
Displacement/calcification of the aorta	5.4	6.5	0.423
Pleural effusion	9.3	12.8	0.051

Note: Bold values indicate significance at *p* < 0.05.

When treating patients in cohort 1, those with head and neck pain were more likely to receive surgical treatment (89.8% vs. 85.2% *p* = 0.001) and correspondingly less likely to undergo medical treatment alone (7.8% vs. 11.4% *p* = 0.005). Significantly more patients with head and neck pain had partial arch replacement compared with those without head and neck pain (47.3% vs. 41.8% *p* = 0.017) ( Table 3 ).

**Table 3:** Treatment modalities for the head and neck pain and no head and neck pain

Variables	Head/neck pain group	No head/neck pain group	*p* -Value
Type of management			
Type A surgical	89.8	85.2	**0.001**
Type A medical	7.8	11.4	**0.005**
Endovascular	1.3	1.8	0.284
Surgery			
Surgery after 24 h	15.5	15.4	0.938
Hours from symptom onset to surgery	6–21	6–21	0.244
Root replacement	34.7	37.9	0.171
Ascending aortic replacement	95.1	93.7	0.202
Complete arch replacement	16.4	17.1	0.704
Partial arch replacement	47.3	41.8	**0.017**
Initial management of β-blockers	56.5	56	0.807

Note: Bold values indicate significance at *p* < 0.05.

While still in the hospital, patients with head and neck pain had higher rates of CVA as a complication (13% vs. 9.9% *p* = 0.016). Regarding overall outcomes ( Table 4 ), mortality among patients with head and neck was 19.5% compared with 23% in those without head and neck pain ( *p* = 0.038). Surgical mortality was slightly lower among patients with head and neck (14.8% and 18.8% *p* = 0.017). Conversely, those patients treated medically showed higher mortality rates among patients with head and neck pain compared with those without (67.7% vs. 51.6% *p* = 0.022).

**Table 4:** Hospital complications and overall outcomes for patients with head and neck pain compared with those without head and neck pain

Variables	Head/neck pain group	No head/neck pain group	*p-* Value
In-hospital complications:			
New neurologic deficit	26.5	24.8	0.343
Cerebral vascular injury	13	9.9	**0.016**
Transient neurodeficit	67.9	60.1	0.085
Coma	4.5	5.5	0.314
Myocardial ischemia	85.1	88.2	0.063
Hypotension	29.4	29.3	0.939
Cardiac tamponade	18.2	18.6	0.799
Mortality:			
Type A overall	19.5	23	**0.038**
Type A surgical mortality	14.8	18.8	**0.017**
Type A medical mortality	67.7	51.6	**0.022**
Cause of mortality:			
Neurologic	10.4	8.2	0.387
Tamponade	4.5	6.8	0.312
Visceral Ischemia	8.4	8.5	0.968
Bleeding	5.2	2.5	0.095
multiorgan failure	9.7	9.7	0.991
Cardiac	7.8	9.1	0.609
Rupture	18.2	18.3	0.984
Unknown	1.9	4.1	0.557

Note: Bold values indicate significance at *p* < 0.05.

In our subgroup analysis of patients with head and neck pain only versus those who had head and neck pain with chest or back, we found that those with head and neck pain only were significantly older (66.4 vs. 61.1 *p* = 0.001). Furthermore, those with head and neck pain only had higher rates of diabetes (19.6% vs. 7.4% *p* = 0.003), atherosclerosis (39.2% vs. 24.1% *p* = 0.018), and previous coronary bypass surgery (12% vs. 1.9% *p* < 0.001) compared with those who also had chest or back pain ( Table 5 ).

**Table 5:** Comparison of baseline demographic risk factors in patients with head and neck pain only compared with those with head and neck pain with chest or back pain

Demographics	Head/neck pain only group	Head/neck pain + chest/back pain group	*p-* Value
Age (overall mean)	66.4 ± 10.6	61.1 ± 14.4	**0.001**
< 40 y	1 (1.9%)	45 (8.3%)	0.167
> 70 y	19 (36.5%)	160 (29.5%)	0.292
Female sex	14 (26.9%)	207 (38.2%)	0.108
White	45 (91.8%)	481 (93.6%)	0.552
African American	1 (2%)	16 (3.1%)	1.000
Asian	1 (2%)	9 (1.8%)	0.601
Family history of aortic disease	0 (0%)	30 (14.2%)	0.084
Risk factors:			
Marfan's syndrome	1 (2%)	25 (4.7%)	0.717
Smoking (current)	5 (26.3%)	68 (33%)	0.551
Hypertension	39 (78%)	378 (71.5%)	0.324
Atherosclerosis	20 (39.2%)	126 (24.1%)	**0.018**
Cocaine usage	0 (0%)	8 (1.6%)	1.000
Bicuspid aortic valve	2 (4.8%)	29 (6.1%)	1.000
Diabetes	10 (19.6%)	39 (7.4%)	**0.003**
Prior surgery:			
Coronary artery bypass grafting	6 (12%)	10 (1.9%)	**< 0.001**
Aortic valve replacement	1 (2%)	13 (2.5%)	1.000
Aortic aneurysm/dissection repair	4 (8.2%)	36 (7%)	0.769

Note: Bold values indicate significance at *p* < 0.05.

At presentation, there were higher rates of coma and altered mental status (32.7% vs. 9.1% *p* < 0.001), syncope (32.7% vs. 14.4% *p* = 0.001), and CVA (18.4% vs. 6.6% *p* = 0.003) in the head and neck pain only group as seen in Table 6 . However, we found no difference in time to presentation or time to diagnosis between the two groups in cohort 2. Furthermore, there was no difference found in management type or extent of surgery between the two groups ( Table 7 ).

**Table 6:** Comparison of presenting symptoms in patients with head and neck pain only compared with those with head and neck pain with chest or back pain

Variables	Head/neck pain only group	Head/neck pain + chest/back pain group	*p-* Value
Presenting within 6 h of symptom onset	28 (90.3%)	324 (86.9%)	0.782
Hours from symptom onset to presentation at initial hospital	1.25 (0.85–4.42)	1.25 (0.75–3.21)	0.600
Hours from symptom onset to diagnosis (avg)	5.62 (3.13–10.13)	5.17 (2.88–14.00)	0.889
Hours from symptom onset to diagnosis:			0.770
0–4 h	10 (34.5%)	135 (40.1%)	
4–24 h	15 (51.7%)	151 (44.8%)	
24 h	4 (13.8%)	51 (15.1%)	
Quality of pain:			
Tearing/ripping	3 (12.5%)	116 (35.3%)	**0.024**
Sharp	11 (44%)	169 (50.8%)	0.515
Pressure	11 (39.3%)	172 (55.3%)	0.103
Burning	5 (20.8%)	42 (15.3%)	0.478
Abrupt onset of pain	43 (84.3%)	477 (90.2%)	0.19
Back pain	0 (0%)	256 (47.2%)	**< 0.001**
Chest pain	0 (0%)	521 (96.1%)	**< 0.001**
Migrating pain	2 (4%)	112 (21.5%)	**0.001**
Coma/altered consciousness	16 (32.7%)	47 (9.1%)	**< 0.001**
Syncope	17 (32.7%)	77 (14.4%)	**0.001**
Cardiovascular accident	9 (18.4%)	34 (6.6%)	**0.003**
Congestive heart failure	3 (6.4%)	23 (4.5%)	0.471
Hypotension/shock/tamponade	14 (28.6%)	126 (24.3%)	0.505
Shock/tamponade	2 (4.1%)	43 (8.3%)	0.412
Shock	1 (2%)	31 (6%)	0.510
Tamponade	1 (2%)	12 (2.3%)	1.000
Murmur of aortic insufficiency	17 (36.2%)	178 (38.5%)	0.751
Any pulse deficit	11 (33.3%)	111 (27.8%)	0.499
Diagnostic imaging findings:			
Arch vessel involvement	23 (56.1%)	169 (41.8%)	0.079
Intramural hematoma (def)	5 (9.6%)	62 (11.6%)	0.663
Periaortic hematoma	7 (14.9%)	103 (23.8%)	0.168
False lumen thrombosis, complete	0 (0%)	34 (10.1%)	0.093
False lumen thrombosis, partial	6 (21.4%)	62 (18.4%)	0.692
Coronary artery compromise	3 (7%)	52 (13.6%)	0.336
Pericardial effusion	23 (45.1%)	203 (42.3%)	0.700
Chest X-ray findings:			
% abnormal	30 (78.9%)	301 (75.3%)	0.612
Wide mediastinum	18 (48.6%)	199 (52.5%)	0.654
Abnormal aortic contour	15 (40.5%)	153 (41.2%)	0.934
Abnormal cardiac contour	9 (24.3%)	76 (20.7%)	0.601
Displacement/calcification of the aorta	4 (10.8%)	20 (5.4%)	0.258
Pleural effusion	1 (2.7%)	35 (9.4%)	0.231

Note: Bold values indicate significance at *p* < 0.05.

**Table 7:** Treatment modality used for those with head and neck pain only compared with those with head and neck pain and chest or back pain

Variables	Head/neck pain only group	Head/neck pain + chest/back pain group	*p-* Value
Type of management:			
Type A surgical	44 (84.6%)	491 (90.6%)	0.169
Type A medical	7 (13.5%)	42 (7.7%)	0.153
Endovascular	0	3 (0.6%)	1
Surgery:			
Surgery after 24 h	9 (20%)	79 (16.2%)	0.51
Hours from symptom onset to surgery	11 (7.75–25.17)	10.64 (6.73–23.63)	0.481
Root replacement	14 (34.1%)	1	0.793
Ascending aortic replacement	42 (93.3%)	443 (94.5%)	0.733
Complete arch replacement	6 (14%)	64 (14.4%)	0.93
Partial arch replacement	20 (44.4%)	20 (46.4%)	0.798
Initial management of β-blockers	21 (47.7%)	268 (53.5%)	0.463

Regarding outcomes, there was a significantly higher percentage of patients with head and neck pain only who had cardiac tamponade while in the hospital (30% vs. 16.6% *p* = 0.018). Regarding mortality rates, patients with head and neck pain only had higher overall mortality (34.6% vs. 19.9% *p* = 0.013) and higher surgical mortality (29.5% vs. 15.3% *p* = 0.014) ( Table 8 ). A logistic regression of mortality was conducted with predictive variables of head and pain, preoperative hypotension, presence of ascending intimal tear, or intramural hematomas, which found that head and neck pain status was not associated with higher mortality after controlling for the other factors ( *p* = 0.36) ( Tables 9–11 ).

**Table 8:** Hospital complications and overall outcomes for patients with head and neck pain only compared with those with head and neck pain with chest or back pain

Variables	Head/neck pain only group	Head/neck pain + chest/back pain group	*p* -Value
In-hospital complications:			
New neurologic deficit	19 (36.5%)	144 (27.5%)	0.167
Cerebral vascular injury	14 (26.9%)	71 (13.8%)	**0.011**
Transient neurodeficit			
Coma	4 (7.7%)	24 (4.7%)	0.312
Myocardial ischemia	4 (8.3%)	63 (12.6%)	0.494
Hypotension	16 (31.4%)	163 (31.5%)	0.989
Cardiac tamponade	15 (30%)	86 (16.6%)	**0.018**
Mortality:			
Type A overall	18 (34.6%)	108 (19.9%)	**0.013**
Type A surgical mortality	13 (29.5%)	27 (15.3%)	**0.014**
Type A medical mortality	5 (71.4%)	28 (66.7%)	1
Cause of mortality:			
Neurologic	3 (16.7%)	13 (12%)	0.701
Tamponade	0	6 (5.6%)	0.593
Visceral ischemia	3 (16.7%)	9 (8.3%)	0.377
Bleeding	1 (5.6%)	6 (5.6%)	1
Multiorgan failure	1 (5.6%)	6 (5.6%)	1
Cardiac	0 (5.6%)	9 (8.3%)	1
Rupture	1 (5.6%)	26 (24.1%)	0.118
Unknown	6 (33.3%)	31 (28.7%)	0.69

Note: Bold values indicate significance at *p* < 0.05.

**Table 9:** Logistic regression model of mortality with independent variables of head and neck pain status, age > 65 years, preoperative hypotension, ascending intimal tear, intramural hematoma

Variables	β-Coefficient	*p* -Value	Risk ratio	95% confidence interval: lower	95% confidence interval: upper
Head and neck and chest or back pain	−0.396	0.359	0.673	0.289	1.568
Age > 65 y	0.806	**0.003**	2.238	1.318	3.802
Preoperative hypotension	1.395	**0.000**	4.037	2.348	6.942
Ascending intimal tear	−0.837	**0.002**	0.433	0.252	0.745
Intramural hematoma	−0.705	0.05	0.494	0.244	1.001
Constant	−1.277	**0.004**	0.279		

Note: Bold values indicate significance at *p* < 0.05.

**Table 10:** Hosmer-Lemeshow test for model fit

Chi-square	Degrees of freedom	Significance
**10.495**	8	0.232

Note: Bold values indicate significance at *p* < 0.05.

**Table 11:** Area under the curve for the logistic regression model

Area under the curve
**0.736**

## Discussion

The acute and lethal nature of acute aortic dissection creates strong impetus for discovering risk factors, symptoms, and other patterns that may speed its diagnosis and decrease its mortality. Complicating this effort are the multiple presenting patterns that occur clinically. 9 13 14 15 16 Head and neck pain is one of these aberrant presentation patterns, which is present in 26.1% of all Type A patients in our database to date.

The pathophysiology of this pain location is not well defined in the literature. Whereas classic angina pectoris is thought to be caused by stimulation of sympathetic afferent nerves around the heart, several studies suggest that the afferent pathway of the vagus nerve may be a cause of cardiac-related head and neck pain. Stimulation of vagal afferents stimulates nerve endings in C1–C3, which corresponds to the receptive field for the neck, jaw, upper arm, and ear. 17 18 19

Another possible mechanism of head pain might be partially explained by our findings that there were significantly more patients with arch vessel involvement and CVA in the head and neck cohort. Because the anterior and posterior circulations of the brain derive from the carotids and the vertebral arteries, respectively, which ultimately obtain blood flow from the aorta, arch vessel involvement has the potential to directly alter flow to the brain. Thus, this would represent a primary cause of the pain rather than referred pain from the heart. Studies have shown that patients with carotid or vertebral dissection can present with headache and neck pain. 20 21

Regarding the higher proportion of head and neck pain found in women in our first analysis ( Table 1 ), it has been shown in two IRAD analyses that women on average present differently than men do with aortic dissection. Women were found to present more frequently with coma and/or altered mental status compared with men. 13 22 Our current data suggest that one reason for higher rates of head and neck pain among women might be more frequent CVA. A 2004 IRAD paper found that 7.9% of women with acute aortic dissection presented with CVA compared with 5.2% men, though this difference was not significant. 13 When analyzing the subgroups in cohort 2, this gender preponderance of head and neck pain in women did not remain. This suggests that though women are more likely to experience head and neck pain overall, those women who have head and neck pain are equally likely to present with chest pain and back pain or with head and neck pain only.

An unexpected finding in our first analysis was the higher rates of chest pain and back pain in the head and neck group along with higher rates of all of the various permutations of pain types such as tearing, sharp, and migrating ( Table 2 ). This may be because “worst-ever pain” in the chest, which radiates to the back, is still the predominant way that patients experience acute aortic dissection, it will remain the most prevalent presentation despite the presence of head and neck pain. 16 23 24 On the other hand, a reason that the non–head and neck group had fewer types of pain may be related to the fact that 15% of patients present with painless dissection, suggesting that there may be aortic dissection patients who are biologically predisposed to feel less pain. 8 25 In fact, in our second analysis, those who experienced isolated head and neck pain were more likely to be older, have more atherosclerosis, and had a higher prevalence of diabetes, all of which were shown to be higher in patients who presented with painless cardiac syndromes. 25 26 27 28 In addition, patients with head and neck pain had higher rates of syncope, coma/altered mental status, syncope, and CVA, which are neurologic phenomena, which may affect the ability to perceive pain, and which may explain why these patients did not report more types of pain. 29

Despite the potential for delayed diagnosis, we found no delay to diagnosis or treatment in either cohort. Furthermore, in cohort 1, *those with head or neck pain had lower overall mortality and lower surgical mortality* . This was unexpected given the atypical presentation. However, given the fact that those with head and neck pain had a higher rate of chest pain and back pain than the group without head and neck pain, we wondered whether the presence of chest and back pain obscured the impact of having head and neck pain. Thus, we conducted a second analysis (cohort 2) with head and neck pain only versus those with head and neck pain with chest or back pain to see the effect of having only head and neck pain (without the influence of chest pain or back pain). We found in the second analysis that there was significantly higher overall and surgical mortality in the head and neck pain only group.

Reasons for the difference in mortality between cohorts 1 and 2 are not clear. Despite those in cohort 1 with head and neck pain having higher rates of the classic symptoms such as chest pain and back pain, we did not see any improvement in time to diagnosis in that group, which would explain their lower mortality. On the other hand, in cohort 2, those who experienced only head and neck pain were older, had higher rates of atherosclerosis, diabetes, and had more coronary artery bypass grafting operations, which represent several important risk factors for mortality. 29 30 31 To analyze this further, we created a logistic regression model for mortality with head and neck pain status, age > 65 years, preoperative hypotension, site of intimal tear and evidence of intramural hematoma as the dependent variables. Ultimately, when controlled for those variables, head and pain neck pain status was no longer significant associated with increased mortality. Given that age > 65 and preoperative hypotension had significant, positive risk ratios in the model, this suggests that these two variables explained some of the increase in mortality.

In conclusion, we have shown that patients with head and neck pain have higher rates of proximal arch involvement and stroke, which adds credence to classic clinical teaching about the association of head and neck pain with arch involvement. Furthermore, patients with head and neck overall tend to have classic dissection symptoms and thus have no delay in diagnosis and also lower mortality compared with those without head and neck pain. However, in our subgroup analysis (cohort 2), we showed that those with isolated head and neck pain tend to have worse comorbidities, more neurologic symptoms on presentation, and worse overall and surgical mortality. This higher mortality may be partially explained by the older age and more preoperative hypotension in the head and neck pain only group. These patients, though a small fraction of our Type A dissection population, represent an area for added vigilance and improvement in our pursuit of better care of acute aortic dissection patients.

Our study has several limitations. IRAD is composed of patients in academic referral centers and may not represent the overall population of aortic dissection patients. In addition, patients with abnormal presentations may pass away prior to diagnosis or transfer to tertiary aortic centers and are thus underrepresented in our population. Furthermore, given our international scope, there is possible variation in treatment patterns, which may complicate outcomes measurements.
